# Anomalous origin of left main coronary artery from the right sinus of Valsalva

**DOI:** 10.1186/s12872-023-03616-x

**Published:** 2023-12-14

**Authors:** Frederick Chua, Kenny Vongbunyong, Deniz Akay Urgun, Roxana Ghashghaei

**Affiliations:** 1 Department of Medicine, University of California, Irvine, USA; 2 Orange, CA USA; 3 Department of Radiology, University of California, Irvine, USA; 4 Department of Medicine, Division of Cardiology, University of California, Irvine, USA

**Keywords:** Anomalous coronary artery, Syncope, Malignant course, Sudden cardiac death

## Abstract

**Background:**

Anomalous coronary arteries are rare congenital variations with cases ranging from asymptomatic to life-threatening. Given the wide variability of coronary anomalies, it is challenging to predict their clinical consequences. Here, we present the ‘malignant’ variant – interarterial course of the left coronary artery between the aorta and pulmonary trunk – given the highest risk of sudden cardiac death among the various coronary anomalies.

**Case presentation:**

Our case presents a 22-year-old male presenting to the emergency department after a syncopal episode that occurred while the patient was driving a motor vehicle. Initial Computed Tomography (CT) of the chest performed as part of the trauma work-up revealed a rare case of an anomalous origin of the left main coronary artery (LMCA) originating from a common ostium with the right coronary artery (RCA). The LMCA was found to have a malignant course, as it was positioned between the aorta and pulmonary artery. Given the high risk of sudden cardiac arrest with this congenital variant, the patient underwent coronary artery bypass grafting.

**Conclusion:**

Anomalous coronary arteries remain the second leading cause of sudden cardiac death in young adult patients. The risk of sudden cardiac death depends on the congenital variant of the anomalous coronary artery as well as the course these vessels take. This case highlights a rare congenital variant featuring both the LMCA and RCA originating from a common ostium, with the LMCA having a malignant course, a variant with the highest risk of sudden cardiac death.

## Background

Anomalous coronary arteries are rare congenital variants with the majority of cases being benign. In some rare variants these anomalies can lead to life-threatening consequences including syncope and sudden cardiac arrest. The anomalous origin of the left main coronary artery from a common ostium with the right coronary artery is an exceedingly rare variation of coronary circulation. When the left main coronary artery traverses between the aorta and pulmonary trunk, it is said to have a ‘malignant’ course, making it prone to compression between the major vessels placing the vessel at increased risk of transient ischemia.

## Case presentation

**Fig. 1:**
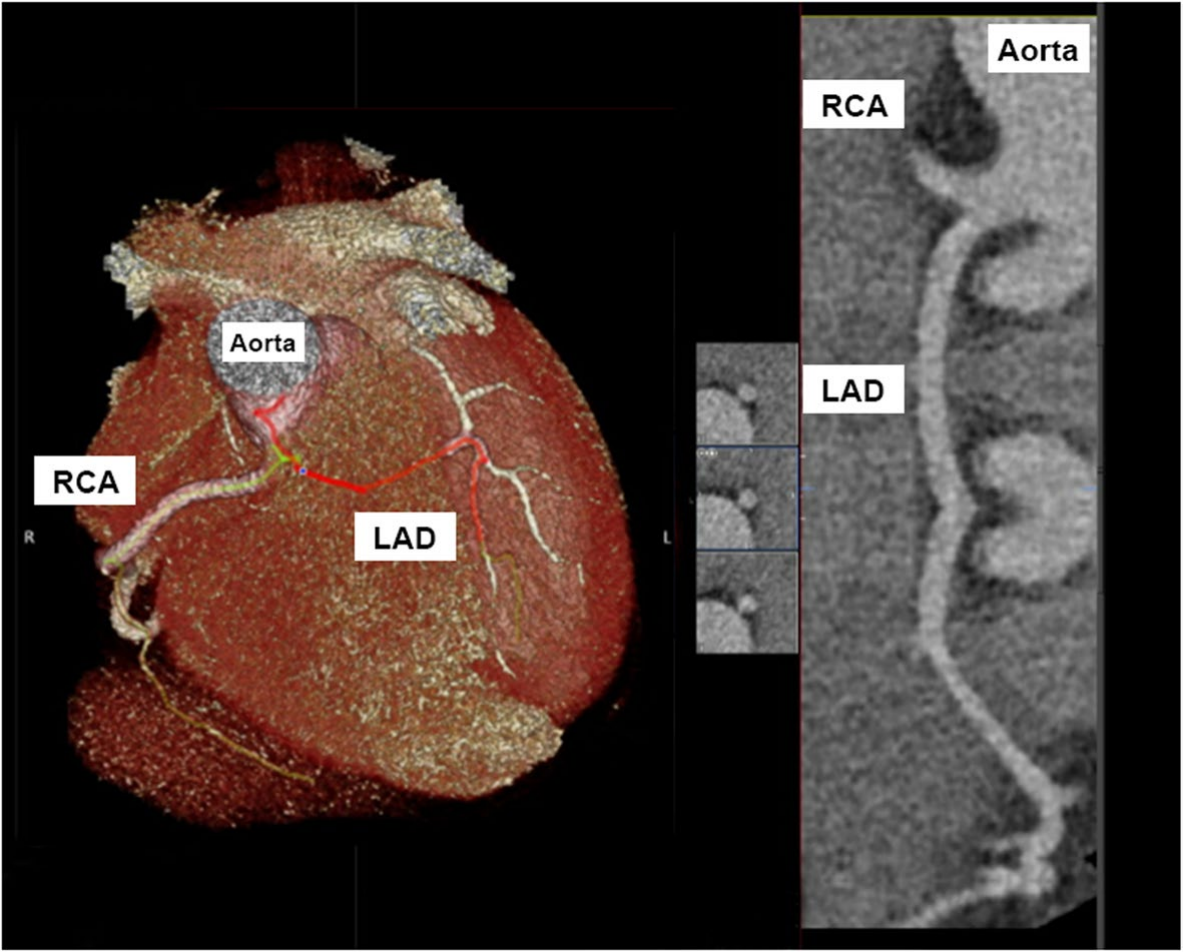
Rendered 3D Computed Tomography showing course of LMCA/LAD

**Fig. 2:**
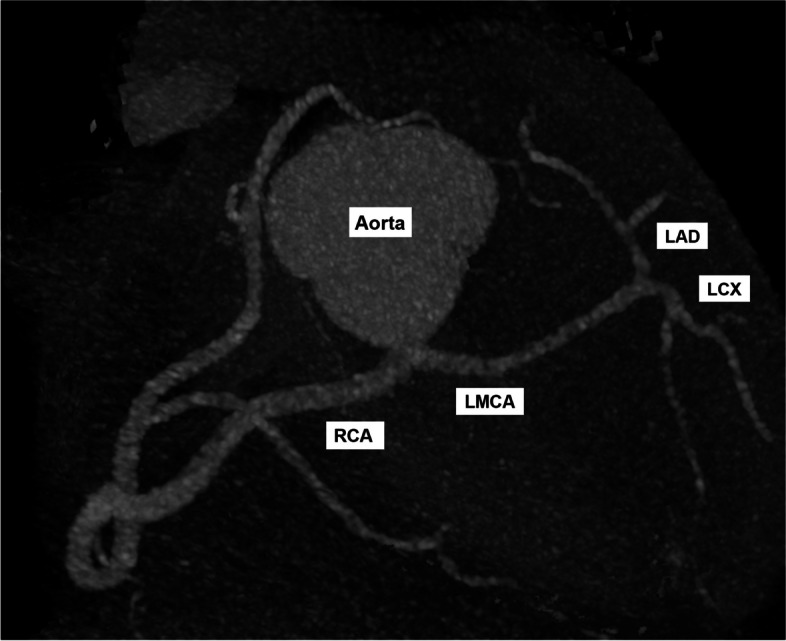
Generated 3D Plane of Heart. LMCA and RCA share a common origin

**Fig. 3:**
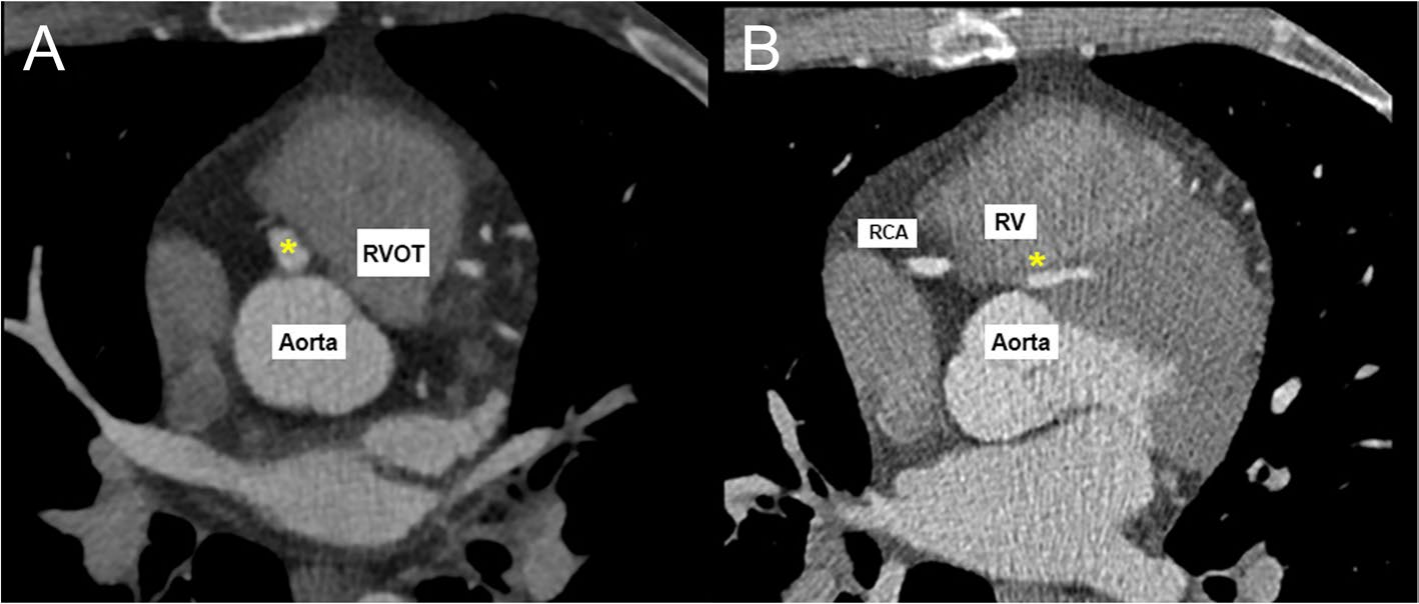
Computed Tomography Axial View. **a** CT Axial view showing common origin of RCA and LMCA (*). **b** CT Axial view showing subpulmonic and ‘malignant’ course of LMCA (*)

A 22-year-old Caucasian man with no prior cardiac history, presented to the emergency department after a syncopal episode while driving a motor vehicle. A full trauma workup was performed including a Computed Tomography (CT) of the chest. The patient was found to have an anomalous origin of the left main coronary artery (LMCA) that originates from a common ostium with the right coronary artery (RCA) at the superior aspect of the Right Sinus of Valsalva (RSV) (Figs. 1 and 2). There is evidence of interarterial course of the LMCA between the aorta and pulmonary trunk (Fig. 3a, b). It is hypothesized that the anomalous course of the patient’s LMCA might be responsible for his syncope, stemming from episodic myocardial ischemia. Cardiothoracic surgery was subsequently consulted. Given how quickly the LMCA dove deep into the septum without an intramural course, surgical unroofing was not performed. Cardiothoracic surgery opted for coronary artery bypass grafting (CABG) with a left internal mammary artery (LIMA) to left anterior descending (LAD) artery anastomosis instead.

## Outcome and follow up

The patient tolerated the CABG well without any complications and was discharged in stable condition. At one month follow-up after discharge the patient continued to recover as expected. He was able to tolerate cardiac rehabilitation well and denied any new syncopal episodes.

## Discussion and conclusions

Among the different variations of congenital coronary artery anomalies, one of the most critical variants features the LMCA originating from the RSV. There are four further sub-types in which LMCA originating from the RSV can be classified as. These include the following: (1) the LMCA passes between the aorta and pulmonary trunk (interarterial), (2) the LMCA passes anterior to the right ventricular outflow tract (prepulmonic), (3) the LMCA courses along the crista supraventricularis in the myocardium or subendocardium and surfaces in the proximal interventricular sulcus (transseptal / intraseptal / subpulmonic), and (4) the LMCA arises to the right of the RCA and circles around posteriorly to the aortic root (retroaortic) [1]. Furthermore, when the LMCA originates from the RSV, the LMCA and the RCA can originate separately or share a common ostium.

Anomalous origin of the left coronary artery from the RSV is rare with an estimated prevalence of 0.02–0.05% [2]. However, second to hypertrophic cardiomyopathy, it remains a leading cause of cardiac death in young athletes. Most patients are asymptomatic and/or the first manifestation of their disease could unfortunately be sudden cardiac death (SCD). Even in those who are symptomatic, they may experience nonspecific symptoms such as palpitations, syncope, and dyspnea on exertion [3]. Multiple hypotheses have been proposed to explain the ‘malignant’ interarterial course. This includes (1) the acute angle of the artery’s “slit-like” ostium which results in narrowing of the vessel and decrease in blood flow, (2) a possible intramural aortic segment leading to endothelial dysfunction and coronary vasospasm, and (3) the interarterial course which is prone to extrinsic compression of the vessel between the aorta and pulmonary trunk. These mechanisms all contribute to myocardial ischemia when a patient exerts themselves, which may result in fatal arrhythmias and SCD [4].

**Fig. 4:**
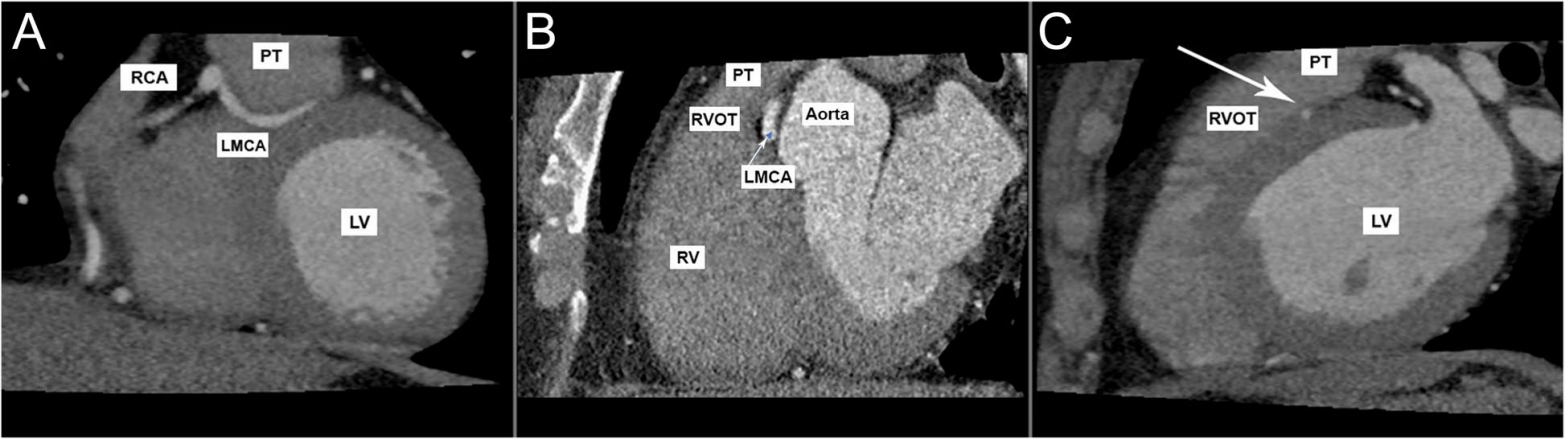
Computed Tomography Coronal and Sagittal View. **a** CT Coronal view demonstrating the common origin of RCA and LMCA with the sub-pulmonic course of LMCA below pulmonary trunk (PT). **b** CT reformatted sagittal view showing LMCA ‘malignant’ course in between PT and Aorta. **c** Computed Tomography Sagittal view showing the deep septal course of LMCA (arrow) within LV wall

In this patient case CT imaging demonstrated a common origin of the RCA and LMCA, with the LMCA taking a sub-pulmonic course (Fig. 4a). The LMCA then has a ‘malignant’ interarterial course traversing between the pulmonic trunk and aortic root (Fig. 4b). Additionally, the LMCA takes a deep septal course within the left ventricle’s wall (Fig. 4c). Therefore, this patient’s specific LMCA course was concerning both extrinsic compression of the LMCA between the aorta and pulmonary trunk and compression of the LMCA from within the left ventricle septal wall during systolic contraction. This unique anatomical course of the LMCA, placed the patient at great risk of sudden cardiac death as the LMCA was vulnerable to compression at multiple points throughout its course, a finding which may also have contributed towards the patient’s syncopal episode.

Additionally, it is important to note that cardiac CT imaging is typically obtained during diastole. This allows for reduced motion at the time of image acquisition and the opportunity to better visualize coronary arteries as they receive blood flow during diastole in the cardiac cycle. Therefore, cardiac CT images can underrepresent the degree of stenosis coronary vessels experience during systole. Similarly, Fig. 3 may underestimate the degree of compression experienced by the LMCA, as the image was captured during diastole and not systole when the left ventricle is contracting.

Due to the high risk of SCD with the interarterial course, there are consensus guidelines from American College of Cardiology (ACC) and American Heart Association (AHA) which recommend surgical intervention even in asymptomatic patients (Class I, Level of Evidence B). In contrast, other variants of anomalous origin of left coronary artery from RSV are considered benign. Patients with benign variants may not be referred for surgery and do not have exercise restrictions. Multiple surgical approaches have been proposed: (1) surgical unroofing for patients with significant intramural length of the anomalous vessel, (2) reimplantation (ostial translocation) when there is little or no intramural segment, and (3) coronary artery bypass graft with mammary artery or saphenous artery conduit when above approaches are technically infeasible [5].

Anomalous origin of left coronary artery is rare, but it is one of the leading causes of SCD in young adults. The interarterial course is the ‘malignant’ variant due to the highest risk of SCD. Multiple proposed mechanisms contribute to ischemia, including the acute angle take-off, possible intramural segment leading to endothelial dysfunction, and external compression of the vessel by the aorta and pulmonary trunk. Therefore, even asymptomatic patients with this variant may be referred for surgery. Surgical options include surgical unroofing of the intramural segment, coronary reimplantation, and coronary artery bypass graft. Increased recognition of the association between the malignant anomalous coronary artery variant and its risk of sudden cardiac death may lead to earlier surgical intervention, reducing the overall incidence of sudden death.

## Data Availability

Not applicable as no datasets were generated or analyzed in this case report.

## Abbreviations

CT: Computed Tomography
LMCA: Left main coronary artery
RSV: Right Sinus of Valsalva
RCA: Right coronary artery
CABG: Coronary artery bypass grafting
LIMA: Left internal mammary artery
LAD: Left anterior descending (artery)
SCD: Sudden cardiac death
LCX: Left circumflex artery
PA: Pulmonary artery
PT: Pulmonary trunk
LV: Left ventricle
